# PATHOGENIC PRE-LEUKEMIC MUTATIONS: RARE IN CLONAL HEMATOPOIESIS BUT ENRICHED IN MYELOID DISEASES

**DOI:** 10.1093/geroni/igae098.1450

**Published:** 2024-12-31

**Authors:** Jing Zhang

**Affiliations:** University of Wisconsin-Madison, Madison, Wisconsin, United States

## Abstract

Clonal hematopoiesis of indeterminate potential (CHIP) refers to the expansion of peripheral blood cells derived from hematopoietic stem cells (HSCs) with at least one somatic driver mutation in healthy elderly individuals. CHIP is strongly linked to aging and confers an increased risk for blood cancers, non-hematological diseases (e.g. cardiovascular disease), and all-cause mortality. In blood cancers, CHIP mutations are associated with initiation of acute myeloid leukemia (AML) and other myeloid diseases and thus considered as pre-leukemic mutations. However, some pathogenic pre-leukemic mutations are rarely found in CHIP patients. We will talk about two of such mutations. The first one is the hot spot G646WfsX12 mutation in the epigenetic regulator ASXL1. This mutation occurs in ~18% of patients with myeloid diseases but almost never found in CHIP patients. Using a knockin animal model (Atm/+), we show that Atm/+ HSCs were only expanded in aged mice but not in young mice. We are investigating if the mutant HSC expansion is caused by reduced immunosurveillance of aged immune cells and/or aged pro-inflammatory stromal cells. The second one is oncogenic NRASG12D mutation, which serves as an initiation or progression mutation in chronic myelomonocytic leukemia. It is shown that one copy of NRASG12D mutation confers bi-modal regulation of HSCs in which it enhances self-renewal in one subset of HSCs but promotes proliferation and exhaustion in another subset of HSCs. We hypothesize that ASXL1G646WfsX12 and NRASG12D mutations belong to the pathogenic pre-leukemic mutations, which easily incorporate additional mutations in leukemogenesis rather than remain stable in CHIP.

